# Saccades Improve Postural Control: A Developmental Study in Normal Children

**DOI:** 10.1371/journal.pone.0081066

**Published:** 2013-11-21

**Authors:** Layla Ajrezo, Sylvette Wiener-Vacher, Maria Pia Bucci

**Affiliations:** 1 Vestibular and Oculomotor Evaluation Unit, ORL Dept., 75019 Robert Debré Paediatric Hospital, Paris, France; 2 UMR 676, INSERM- Université Paris 7, Hôpital Robert Debré, Paris, France; University of California, Merced, United States of America

## Abstract

**Introduction:**

Dual-task performance is known to affect postural stability in children. This study focused on the effect of oculomotor tasks like saccadic eye movements on postural stability, studied in a large population of children by recording simultaneously their eye movements and posture.

**Materials and Methods:**

Ninety-five healthy children from 5.8 to 17.6 years old were examined. All children were free of any vestibular, neurological, ophtalmologic and orthoptic abnormalities. Postural control was measured with a force platform TechnoConcept®, and eye movements with video oculography (MobilEBT®). Children performed two oculomotor tasks: fixation of a stable central target and horizontal saccades. We measured the saccade latency and the number of saccades during fixation as well as the surface, length and mean velocity of the center of pressure.

**Results:**

During postural measurement, we observed a correlation between the age on the one hand and a decrease in saccade latency as well as an improvement in the quality of fixation on the other. Postural sway decreases with age and is reduced in the dual task (saccades) in comparison with a simple task of fixation.

**Discussion - Conclusion:**

These results suggest a maturation of neural circuits controlling posture and eye movements during childhood. This study also shows the presence of an interaction between the oculomotor system and the postural system. Engaging in oculomotor tasks results in a reduction of postural sway.

## Introduction

Postural control has been considered as an automatic system but recent studies suggest that several processes are involved in the regulation of posture [1]. For instance, attention is involved in postural stability and its mobilization could depend on many factors, such as availability of sensory information, type of task and age of participants [2], [3]. Several studies explored the effect of a dual task on postural stability and their results are quite controversial, however those different results could be due to different types of secondary tasks used and/or different postural parameters measured. Blanchard et al. [4] studied the effects of a cognitive task on balance in children (between 8 and 10 years old) and reported an improvement in postural stability when children are counting backward compared to when they are looking at an image or reading a text. In contrast, Schmid et al. [5], using a similar cognitive task (mentally counting backwards in steps of two), showed a decrease of postural stability in 9-year-old children. The group of Olivier [6] reported an increase in postural sway in children (mean age 7.3±0.2 years) in comparison with adults (mean age 25.7±2.25 years) when performing a dual-task (modified Stroop test). In the same way, Laufer et al. [7] observed poor postural control in 5-year-old children when they had to name objects that appeared consecutively on a screen. All these results suggest a significant interaction between cognitive processes and balance capabilities. In another study, Olivier et al. [8] explored the interference between postural control and a secondary cognitive task (congruent and non-congruent Stroop conditions) in children versus adults, on a global population of 46 subjects from 7 to 25 years old. They found a non-linear decrease in postural sway during childhood, whatever the level of complexity of the cognitive task, and a maturation level of attention reached at around 11 years of age. These authors and Palluel et al. [9] suggested that two independent attentional mechanisms could exist, one for controlling posture and the other responsible for the secondary cognitive task. These two mechanisms could interfere with each other depending on the difficulty of the dual-task (cognitive and postural).

Recently, Scharli et al. [10] measured the surface of the centre of pressure (COP) as well as head movements in sixty subjects (aged from 5 to 11 years old and young adults) during quite stance with eyes closed, during fixation of a target and during shifting of the gaze between two dots. They found that the surface of the COP decreased with age, suggesting an improvement of postural control from five to eleven years of age. Importantly, 5-year-olds children showed in the gaze shift condition more head movements and poor postural stability than other groups of subjects. These authors suggested that such excessive head movements, particularly during gaze shifts in the group of 5-year-olds children could be a primary cause of their poor postural stability. A subsequent study of the same group [11] compared the surface area of the COP and head movements in children population versus an adult group while subjects were fixating a dot and were watching a movie. They found a decrease of head rotation and of the COP displacement when the age increases in both conditions; for children, the surface of the COP was larger in the watching-a-movie condition with respect to the dot-fixation condition while for all subjects, independently from their age, head movements were greater in the watching-a-movie condition with respect to the dot-fixation condition. All these data together suggest that head instability is linked to gaze shift and that, for children, such instability is an important limiting factor in postural control.

In adults, the effect of eye movements on postural control has been under investigation for long time. Uchida et al. [12] found that even if saccadic eye movements were performed in the dark, they reduced postural sway. In another study, White et al. [13] did not show any effect on posture when voluntary saccadic eye movements were performed standing on one foot. However, they reported some postural change when visual surrounding was moving. Recently Laurens et al. [14] pointed out the effect of the visual background on the postural control. They showed that a static visual background had a stabilizing effect independent from whether the subjects are fixating or making eye movements. Glasauer et al. [15] observed during Tandem Romberg position an increase of postural instability when adult subjects were making pursuit eye movements with respect to a simple target fixation. Stoffregen et al. [16] explored head and torso sway changes while adult subjects were performing saccades or fixating a target with both eyes closed and opened. These authors found a reduced sway while subjects were performing saccades relative to when they were fixating a target in the condition both eyes opened; sway was also smaller in the saccadic condition with eyes opened with respect to the same condition with eyes closed. Furthermore, with both eyes closed, head sway was less variable during saccades than during fixation task. These findings are in line with the hypothesis of a functional integration of postural control with visual performance. In other words, postural control could be modulated in order to execute saccadic eye movements in a correct way. In line with such hypothesis, Rougier and Garrin [17] compared the effect of blinks and saccadic eye movements on postural stability in adults. They found that blinks did not change postural sway; in contrast horizontal and vertical saccades reduced postural sway. These authors suggested that postural control could be modulated in order to facilitate the performance of the secondary (oculomotor) task as suggested by the supra-postural concept of Stoffregen et al. [16]. Note however, that the controversial results could be due to the varying experimental conditions used in the different studies, such as the type of postural test (bipodal or unipodal postion) or characteristics of eye movements (pursuits or saccades amplitude varying from 4 to 40°, horizontally or vertically directed).

In the present study, we wanted to explore the effect of an oculomotor task on postural stability in a large population of children. We compared the effect of saccadic eye movements and fixation on postural stability. Since attention is known to be involved in the execution of saccadic eye movements [18], [19], our driven hypothesis is that a task including saccades should modify postural stability given that several cortical structures (i.e., frontal, parietal, occipital) and brainstem areas (as the paramediane pontine reticular formation and superior colliculus) play an important role in both the performance of saccadic eye movements and postural control [20]–[22]. Based on this, we could expect to find an interference between oculomotor and postural control while saccades and postural tasks are executed in a dual task.

Few studies have explored the effect of saccades on postural stability in children. Our group compared postural performance in dyslexic versus non dyslexic children (mean age 10.5±1 years) while performing saccades or reading a text [23]. We found that saccades improved postural stability in comparison with reading; this could be due to the fact that during reading saccades are done together with a cognitive activity (namely word comprehension). Furthermore, we showed that saccades improved postural control in a population of 18 healthy children as well as in children with strabismus with matched age (from 6.8 to 16 years old) in comparison with conditions in which children had to fixate a stable target [24]. We suggested that the postural improvement observed in a dual task (saccades) vs. a simple task (fixation) might be due to the fact that postural control becomes more automatic during saccadic eye movements. All these findings are in line with the U-shaped non linear interaction model described by Lacour et al. [25] showing that a secondary task performed during a postural task could increase or decrease postural stability depending on its complexity.

Note, however, that in all of these studies dealing with eye movements and postural control in children, eye movements performances were never analysed. The novelty of the present study is that we recorded simultaneously both eye movements and posture in a large population of children (95 subjects from 5.8 to 17.6 years old) and eye movements as well as postural parameters had been analysed. According to previous work on developmental aspects of saccadic performances [26] and postural capabilities [27] we expected to find, as a function of age, a different degree of attentional resources allocated for performing saccadic eye movements as compared to a fixation task.

## Materials and Methods

### Subjects

Ninety-five children (aged 5.83 to 17.58 years) participated to the study. For better presentation children were divided in five groups depending on their age (see Table 1): Group 1 composed of 19 children aged 5–7 years (mean age: 6.44±0.08 years); Group 2 composed of 22 children aged 7–9 years (mean age: 7.62±0.13); Group 3 composed of 16 children aged 9–11 years (mean age: 9.58±0.12); Group 4 composed of 20 children aged 11–14 years (mean age: 12.19±0.16) and Group 5 composed of 18 children aged 14–18 years (mean age: 15.04±0.20). ANOVA test on mean age showed a significant difference between all these groups (F_(4,90)_ = 571.75 p<0.001).

**Table 1:** Composition of the age-related groups of children with the mean, standard error.

Groups	Age range (years)	Mean age ±SE	Number of children	Number of girls	Number of boys
1	5–7	6.44±0.08	19	9	10
2	7–9	7.62±0.13	22	10	12
3	9–11	9.58±0.12	16	6	10
4	11–14	12.19±0.16	20	14	6
5	14–18	15.04±0.20	18	12	6

All subjects underwent an ophthalmologic, orthoptic, neurological and vestibular evaluation. Details on these clinical findings are given below.

The investigation adhered to the principles of the Declaration of Helsinki and was approved by our institutional Human Experimentation Committee (Comité de Protection des Personnes CPP Ile de France V, Hôpital Saint-Antoine). Informed written consent was obtained for each subject and from the children's parents after careful review of the experimentation with the participants.

### Ophthalmologic and orthoptic evaluation

All subjects had normal values for ophthalmologic and orthoptic examination (Table 2 reports the clinical data obtained). The corrected visual acuity was normal (≥20/20) for all subjects. All subjects had normal binocular vision (mean value 58.16±18.34 s of arc), as evaluated with the TNO random dot test. The near point of convergence (NPC) was normal for all subjects (mean value 2.06±2.31 cm). Heterophoria (i.e. the latent deviation of one covered eye when the other is not covered) measured by using the cover-uncover test at near distance (30 cm) was normal for all subjects (mean value −3.22±3.62 pD). Fusional amplitudes of convergence (mean value 37±8.21 pD) and divergence (mean value 16.72±2.96 pD) were measured at near distance (30 cm) by using a base-in and a base-out prism bar. Divergence amplitude was measured twice, before and after the convergence measure, in order to evaluate accommodative spasm [28]. Subjects had no accommodative spasm. None showed any paresis or strabismus.

**Table 2:** Clinical characteristics of the five groups of children examined.

Subjects (yrs)	TNO (s of arc)	PPC (cm)	Phoria (pD)	Convergence (pD)	Divergence (pD)
**Group 1** (6.44±0.08)	55±12.58	2.00±2.24	−3.89±3.36	40.79±5.07	17.74±2.66
**Group 2** (7.62±0.13)	66.82±22.55	2.18±2.94	−3.55±3.90	39.55±5.96	17.91±1.31
**Group 3** (9.58±0.12)	58.75±19.62	1.56±1.55	−3.25±3.26	37.19±6.82	16.38±3.67
**Group 4** (12.19±0.16)	57.00±19.22	2.40±2.33	−2.60±3.50	33.40±9.90	16.65±2.91
**Group 5** (15.04±0.20)	51.67±12.49	2.06±2.26	−2.78±4.18	33.72±10.00	14.56±3.05

Mean values of: binocular vision (Stereoacuity test, TNO measured in seconds of arc); near point of convergence, NPC measured in cm; Heterophoria at near distance measured in prism diopters; negative values indicate exophoria and positive values indicate esophoria; Vergence fusional amplitudes (divergence and convergence) at near distance measured in prism diopters.

We noticed that convergence and divergence values were different between the older and the younger children but all these values were still normal. The ANOVA showed a significant group effect only for convergence (F_(4,90)_ = 3.57, p<0.01) and divergence (F_(4,90)_ = 4.51, p<0.002). Post hoc comparisons showed that Group 4 had a lower mean value of convergence than Group 1 (p<0.004) and Group 2 (p<0.01), and that Group 5 had a lower mean value of convergence than Group 1 (p<0.007) and Group 2 (p<0.02). Post hoc comparisons showed that Group 5 had a lower mean value of divergence than Group 1 (p<0.0007), Group 2 (p<0.0002) and Group 4 (p<0.02). Group 5 had an almost significant lower mean value of divergence than Group 3 (p = 0.057).

### Neurological, hearing and Vestibular evaluation

The clinical examination included a neurological examination, a hearing examination to assess the function of the inner ear (tonal and speech audiometric techniques) and a vestibular evaluation including Hamalgyi's test (to evaluate clinically the function of the semicircular canals (see Legrand et al. [29] for more details). The results of all these tests were normal for all subjects.

### Visual tasks

Two visual tasks were designed and performed in separate sessions: fixation and saccades. The stimuli were presented on a flat PC screen of 22″, its resolution was 1920×1080 and the refresh rate was 60 Hz. Stimuli were presented on the screen at 15°.

#### Fixation

subjects had to fixate a white filled circle subtending a visual angle of 0.5° appearing in the center of the screen and switched on during 25.6 sec. Note that even if this visual task is quite a difficult task, requiring precise active stabilization of the eyes and attention (Legrand et al. [30]), it is usually used as a control task for postural measures (see articles cited in the Introduction).

#### Saccades

horizontal, visually-guided saccades were elicited using a simultaneous paradigm. Subjects had to fixate a green filled square on a period randomly ranging between 2000 and 3500 ms, then the central target disappeared and a red filled square on the left or on the right side of the screen was switched on for 1000 ms. The central fixation target then reappeared, signalling the beginning of the next trial.

A total of 24 saccades of 20° of amplitude were elicited: 12 saccades were centrifugal and the other 12 were centripetal and were randomly presented. We analyzed only the centrifugal saccades. While performing the visual tasks, the subject was standing on a platform and both eye movements and posture were recorded simultaneously.

### Postural recording

To measure postural stability, we used a platform (principle of strain gauge) consisting of two dynamometric clogs (Standards by Association Française de Posturologie, produced by TechnoConcept, Céreste, France). The excursions of the center of pressure (COP) were measured during 25.6 seconds; the equipment contained an analog-digital converter of 16 bits. The sampling frequency of the COP was 40 Hz.

Postural measurements were performed in Standard Romberg condition: the heels were placed four centimeters apart and feet positioned symmetrically with respect to the participant's sagittal axis at a 30° angle. Before running postural measure for each child, the program asked to add the weight, the size and the shoe size. Postural analysis takes in account these individual data.

For each visual task two postural recordings were done successively. The order of the visual tasks varied randomly across subjects. The experimental sessions took place in a dark room to avoid that child could fixate other stimuli. Subjects were placed 60 cm away from the screen, where visual tasks were presented at eye level. Subjects were asked to stand without moving their body and with their arms along their body. Children were asked not to move their head during the visual tasks.

### Eye movement recording

During the postural recording, eye movements (fixation or horizontal saccades) were recorded binocularly by a non-invasive system using infrared camera and mirror; horizontal and vertical eye position were recorded independently and simultaneously for each eye with the Mobile EyeBrain Tracker (Mobile EBT®, e(ye)BRAIN, www.eye-brain.com), an eye-tracking device CE approved for medical applications. Recording frequency was set up to 300 Hz.

Calibration was done at the beginning of eye movement recordings when subject was already on the platform. The calibration consisted of a succession of red points (diameter 0.5 deg) presented randomly on the screen following a grid of 13 points. The calibration was calculated for a period of fixation of 250 ms for each point (see Lions et al. [31] for details). The task started immediately after the calibration.

### Data processing

To quantify the effect of visual tasks on the postural performance, several parameters of the platform recording were analyzed: the surface area, the length and the mean speed of the center of pressure (CoP). The surface area and the length permit efficient measurement of CoP spatial variability [32]. The surface of CoP corresponds to an ellipse with 90% of CoP excursions. The length of CoP is the path of the center of pressure. These two postural parameters are uncorrelated; indeed the inner surface of the same length may be different [33], [34]. The mean speed represents a good index of the amount of neuromuscular activity required to regulate postural control [35], [36].

Eye movements from the dominant eye of each subject were analysed. During the fixation task, the number of saccades with amplitude ≥±2° was counted. It is well known that micro saccades are normally smaller than such amplitude [37].

For each saccade recorded during the horizontal saccadic task, we examined the latency of the saccades in milliseconds (i.e. time needed to prepare and trigger the saccades). The MeyeAnalysis© software (provided with the eye tracker, see www.eye-brain.com) was used to determine automatically the onset and the end of each saccade by using a ‘built-in saccade detection algorithm.’ All detected saccades are verified by the investigator and corrected or discarded as necessary [26].

### Statistical analysis

Analyses of variance (using the ANOVA test) were performed with the different groups of children as between-subject factor and the individual means of eye movements and postural parameters as within-subject factors (STATISTICA®).

Post hoc comparisons were made with the Fischer's least significant differences (LSD) test used to explore further and compare the mean of one oculomotor task or postural position with the mean of another. The effect of a factor was considered as significant when the p-value was below 0.05.

## Results

### Eye movements

**Figure 1:**
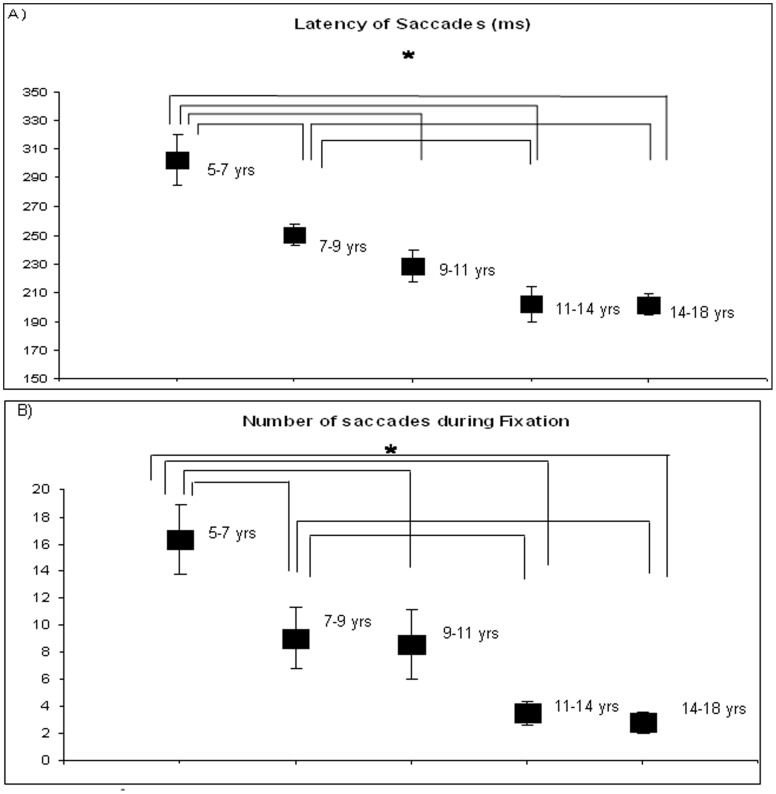
Eye movements executed during postural task for the five groups of subjects. **Figure 1A**: Mean values of latency of saccades (in milliseconds) during postural task. Vertical bars indicate the standard error. Asterisks indicate that the value is significantly different (p<0.003). **Figure 1B**: Mean values of number of saccades during fixation during postural task. Vertical bars indicate the standard error. Asterisks indicate that the value is significantly different (p<0.02).

Figure 1A shows the mean latency of saccades (in milliseconds) for each group of subjects. The ANOVA showed a significant age effect (F_(4,90)_ = 12.68, p<0.001) indicating that saccade latency decreased as age increased. Post hoc comparisons showed that the mean latency of saccades for Group 1 was significantly longer than the mean latency of saccades for the other groups of subjects (p<0.001) and the mean latency of saccades for Group 2 was significantly longer than the mean latency of saccades for Group 4 (p<0.002) and for Group 5 (p<0.003).

Figure 1B shows the mean number of saccades during fixation for each group of subjects. The ANOVA showed a significant age effect (F_(4,90)_ = 3.28, p<0.02): the number of saccades during fixation decreased as age increased. Post hoc comparisons showed that the mean number of saccades during fixation for Group 1 is significantly higher than the mean number of saccades during fixation for the other groups of subjects (p<0.02); in the similar way the mean number of saccades during fixation for Group 2 is significantly higher than the mean number of saccades during fixation for Groups 4 and 5 (p<0.02).

### Postural task

**Figure 2:**
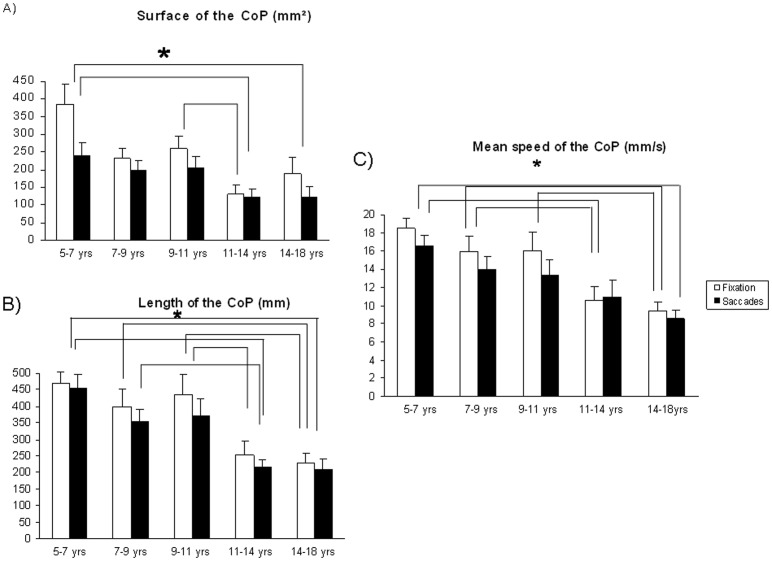
Postural parameters recorded during fixation and saccades for the five groups of subjects. **Figure 2A**: Mean values of the surface of the CoP (in mm^2^) during fixation and saccades. Vertical bars indicate the standard error. Asterisks indicate that the value is significantly different (p<0.05). **Figure 2B**: Mean values of the length of the CoP (in mm) during fixation and saccades. Vertical bars indicate the standard error. Asterisks indicate that the value is significantly different (p<0.02). **Figure 2C**: Mean values of the mean speed of the CoP (in mm/s) during fixation and saccades. Vertical bars indicate the standard error. Asterisks indicate that the value is significantly different (p<0.03).

Figure 2A shows the mean surface of the CoP for each group of subjects during fixation and saccade tasks. The ANOVA showed a significant age effect (F_(4,90)_ = 3.40, p<0.01). Post hoc comparisons showed that the mean value of the surface of the CoP for Group 1 was significantly larger than the mean value of the surface of the CoP for Group 4 (p<0.001) and Group 5 (p<0.005), and the mean value of the surface of the CoP for Group 3 was significantly larger than that of Group 4 (p<0.05).

The ANOVA showed a significant effect of the visual task (F_(4,90)_ = 13.60, p<0.001) indicating that the mean value of the surface of the CoP increased significantly during fixation than during saccades. The ANOVA failed to show a significant interaction between groups and tasks (F_(4,90)_ = 2.12, p = 0.08).

Figure 2B shows the mean value of the length of the CoP for each group of subjects during fixation and saccade tasks. The ANOVA showed a significant age effect (F_(4,90)_ = 8.41, p<0.001). Post hoc comparisons showed that the mean value of the length of the CoP for Group 5 was significantly lower than the mean value of the length of the CoP for Group 1 (p<0.001), Group 2 (p<0.003) and Group 3 (p<0.003), and the mean value of the length of the CoP for Group 4 was significantly lower than that of Group 1 (p<0.001), Group 2 (p<0.002) and Group 3 (p<0.002). The ANOVA showed a significant effect of the visual task (F_(4,90)_ = 5.82, p<0.02): the mean value of the length of the CoP was significantly larger during fixation than during saccades. The ANOVA failed to show a significant interaction between groups and tasks (F_(4,90)_ = 0.78, p = 0.54).

Figure 2C shows the mean value of the mean speed of the CoP for each group of subjects during fixation and saccade tasks. The ANOVA showed a significant age effect (F_(4,90)_ = 5.70, p<0.001). Post hoc comparisons showed that the mean value of the mean speed of the CoP for Group 5 was significantly lower than the mean value of the mean speed of the CoP for Group 1 (p<0.001), Group 2 (p<0.003) and Group 3 (p<0.008), and the mean value of the mean speed of the CoP for Group 4 was significantly lower than that of Group 1 (p<0.001) and Group 2 (p<0.03).

The ANOVA showed a significant effect of the visual task (F_(4,90)_ = 11.30, p<0.001), the mean value of the mean speed of the CoP was significantly greater during fixation than during saccades. The ANOVA failed to show a significant interaction between groups and tasks (F_(4,90)_ = 1.67, p = 0.16).

## Discussion

The main findings of this study are as follows: (i) During postural task, the latency of saccades decreases with the age of the subjects and the quality of fixation improves with age; (ii) Postural stability improves with age during simple tasks (fixation) as well as in dual tasks (saccades); (iii) During dual tasks (saccades), postural stability improves with respect to simple tasks (fixation). These findings are discussed individually below.

### Latency of saccades and quality of fixation during posture

Our study shows that the latency of saccades while performing postural measure decreases with increasing age between 5.8 and 17.6 years old. This result is in line with previous studies of our [26] and other groups [38]–[40] examining latency of saccades while children were seated on a chair with their face resting on a forehead and chin support. The shortening of saccade latency could be associated to the cortical maturation during childhood. Luna et al. [41] reported that cortical circuits responsible for the preparation of saccades are not completely developed in children. Indeed, it is assumed that several processes take place during saccade latency, such as the shift of visual attention to a new target, the disengagement of ocular motor fixation and the computation of new parameters [42], [43], and these processes involve different cortical and subcortical areas [21].

Furthermore, our data showed a turning point at about 12 years old, after which saccade latency does not change significantly, as reported in previous studies cited above, which could indicate a critical step of maturation of the cortical area involved in oculomotor control. This could be due to the reduction of grey matter in the frontal and temporal areas through childhood [44], [45].

In line with this thinking, the quality of fixation, marked by a low frequency of saccades, also improved with age. Indeed, recall that in our fixation task, children were asked to avoid making saccades; consequently the less saccades are done during this task, the more correctly the fixation task is executed. The quality of fixation is rarely reported in children studies. Our data show that fixation improves with age given that the number of saccades during fixation is significantly reduced in the older group of subjects tested (14–17 years old), compared to the younger groups (6–10 years old). Such finding is in line with Munoz et al. [46], showing improvement of fixation ability from 5 to 15 years of age. Other studies examining fixation capabilities in a population of children should be conducted to confirm these results.

### Postural stability improves with age

Our results showed an improvement of postural stability with age in the presence of either a dual or a simple task. This finding is in agreement with the recent review of Assaiante [27] showing that the development of postural control is a continuous process until adolescence. Assaiante & Amblard [47] reported that the adult-like balance control strategy starts being adopted at around 8 years. The recent reports of Scharli et al. [10], [11] are also in line with this hypothesis showing a reduction of head movements with age. Blanchard et al. [4] studied the effect of a dual-task condition in children between 8 and 10 years old and reported that the length of the CoP was larger to that of adults. Similarly, Olivier et al. [6] showed an increase in postural sway in children compared with adults when performing a single postural task or a dual task. Olivier et al. [48] showed in a population of 55 subjects that maximal amplitude and mean velocity of CoP decreased between 4–5 and 6–7 years, reached a plateau around the ages of 6–11 years and decreased again between 10–11 years. These authors suggested that the period of 8–11 years can be considered as a critical period resulting from a better integration of sensory information. Indeed, Baumberger et al. [49] recorded postural stability during optical flow exposure on the ground in a population of 56 subjects from 7 to 11 years old and 12 adult subjects, and they found that the period of 8–11 years old is critical for reaching the maturity of sensorimotor coordination. This critical period occurs at the same time as the adult-like head–trunk coordination [47].

For all the parameters measured (surface, length, mean speed of CoP), we found a similar trend, that is a period between 7 and 9 years old with insignificant changes in postural control parameters. After 9 to 12 years old, we found a statistically significant improvement of this postural stability, corresponding to the maturation level of postural stability reached at 12 years old, as described in previous studies cited above.

Finally, it should be noted that in the present study, COP data have been analyzed in the spatial domain; however, other types of analysis can be done in the temporal domain.

According to our findings Hong et al. [50] showed that the magnitude of sitting postural sway variability decreased with age. The new finding of the Hong's study is that the postural dynamic analysis reported a significant increase in relative entropy of sway motion (in across the x and y axes) in young adults with respect to 6 year-old children. These authors suggested that the changes in the dynamic of sitting postural sway in young children with respect to adults could be not only due to the increasing age of subjects but also to their motor experiences.

Further studies dealing with both temporal and spatial analysis of the COP will be needed in a large population of children and adults in order to improve our understanding of such issue.

### Saccades improves postural stability

Our results showed that performing saccades improves postural stability with respect to a simple task (fixation) regardless of age. Our observation is in agreement with Uchida et al. [12], with Rougier & Garin [17], and with Stoffregen et al. [16] who showed in adult subjects an improvement of postural control during saccadic eye movements. Similarly, our group [23], [24] reported an improvement of posture while performing saccades, for healthy children as well as for children with dyslexia and strabismus. These results are in line with the U-shaped non-linear interaction model described by Lacour et al. [25], which shows that performing a secondary task during a postural task could prevent attention from being focused on postural stability, leading to a reduction of postural sway (automatic attentional system). In other words, such improvement might be due to the fact that postural control could become more automatic. Alternatively, as suggested by the works of Stoffregen et al. [16], [51] the decrease of the postural sway could allow the child to better perform the saccadic task. The relationship between postural control and saccadic eye movements could allow a good saccadic performance by adaptive postural changes.

Finally this study also shows an interaction between the oculomotor and the postural system, according to the fact that the same structures of the central nervous system play an important role in postural control as well as in programming and executing saccadic eye movements [21].

According to Campos et al. [52], postural improvement by age could be also due to perceptual, motor and socio-emotional capabilities arising from experience that naturally occurs during the development of children. We suggest that the evolution of posture with growing age could be due to neural plasticity allowing children to improve their postural stability with daily experience. This hypothesis is in agreement with the thinking that the brain undergoes continuous change in response to modifications of internal and external inputs [53].

## Conclusion

During childhood (from the age of 6 to adolescence) our data show that postural control as well as saccades and fixation improve with age. More specifically, we show a step of maturation of postural control at around 9–12 years old. Postural sway reduces as age increases. Performing saccadic eye movements results in a reduction of postural sway.
